# Medical Items

**Published:** 1911-02

**Authors:** 


### MEDICAL ITEMS

### THE DIFFERENCE BETWEEN MORPHINE AND CODEINE AND HEROIN.

A short time ago the Board of Health of the city of New York, promulgated an ordinance providing that "No cocaine or salt of cocaine, and no morphine or salt of morphine, either alone or in combination with other substances, shall be sold at retail by any person in the city of New York, except upon the prescription of a physician." Immediately every druggist in the city stopped the sale of all preparations containing any derivative of opium and raised such a furore, that the Acting Commissioner of the Board of Health felt called upon to explain what every druggist ought to have known, viz: that "Heroin and Codeine are not salts of morphine, and therefore are not included in the proscribed list."

In order to make this matter perfectly clear, the following on the subject of opium is submitted for the information of the many who have been laboring under the misapprehension that Codeine and Heroin are salts of opium or of morphine.

Opium, besides wax, fat, glucose, gum, pectin, resin, etc., contains about 20 alkaloids, among them being Morphine, Codeine, Thebaine, Naceine, Papaverine, Pseudo-morphine, Narcotine, all occurring in varying amounts according to the grade of opium. One might equally as well say that Acetanilid and Diamond Dyes have similar therapeutic effects, because both are derived from coal tar. Heroin, as is well known to every druggist, is a synthetic preparation and is not an alkaloid of opium. There are no salts of opium; there are active principles or alkaloids from which, by the addition of acids, salts are formed, which become, not salts of opium, but salts of morphine, salts of codeine, etc. All chemists know this and all druggists probably know it, but

